# Comparative Genomic Hybridization Identifies Virulence Differences in *Streptococcus suis*

**DOI:** 10.1371/journal.pone.0087866

**Published:** 2014-02-04

**Authors:** Han Zheng, Ruiting Lan, Xiao Zheng, Zhigang Cui, Zhijie Liu, Xuemei Bai, Shaobo Ji, Marcelo Gottschalk, Jianguo Xu

**Affiliations:** 1 Collaborative Innovation Center for Diagnosis and Treatment of Infectious Diseases, State Key Laboratory for Infectious Disease Prevention and Control, National Institute for Communicable Disease Control and Prevention, Chinese Center for Disease Control and Prevention, Changping, Beijing, China; 2 School of Biotechnology and Biomolecular Sciences, University of New South Wales, Sydney, New South Wales, Australia; 3 Groupe de Recherche sur les Maladies Infectieuses du Porc, Faculté de médecine vétérinaire, Université de Montréal, Montréal, Québec, Canada; State Key Laboratory of Pathogen and Biosecurity, Beijing Institute of Microbiology and Epidemiology, China

## Abstract

*Streptococcus suis* is an important zoonotic pathogen. However, identification of virulent *S. suis* strains is complicated because of the high diversity of the species. Here we evaluated the genetic difference among *S. suis* strains using comparative genomic hybridization (CGH) and virulence variation *in vivo* and *in vitro*. We showed that different clades differed in their ability to activate TLR2/6 *in vitro* and their capacity to induce cytokine production *in vivo* as well as their resistance to phagocytosis and survival *in vivo*. Our data showed the *S. suis* strains tested can be classified into three groups having differing levels of virulence: epidemic and highly virulent strains were clustered into clade Ia (epidemic and highly virulent group, E/HV group), virulent strains were clustered into clade Ib (virulent group, V group), and intermediately or weakly virulent strains were clustered into other clades (intermediately or weakly virulent group, I/WV group). Our study provided further insight into the genomic and virulence variation of *S. suis*.

## Introduction

*Streptococcus suis* is a major swine pathogen. It causes a wide variety of diseases in pigs, including meningitis, septicemia, and endocarditis [1], [2]. Virulence among *S. suis* strains is highly variable, adding to the complexity of understanding pathogenic mechanisms. Further, virulence of some strains may also vary depending on the experimental animal models or methods were used, e.g. 89-1591 (ST25)[3], [4], [5], [6], S735[7], [8].

Previously, we performed a comparative genome hybridization (CGH) study to assess the genomic variation of 31 *S. suis* strains from different clinical sources using a highly pathogenic strain from a human patient: GZ1 (serotype 2, ST1, ST1 Complex) as the reference [9]. We found the *S. suis* genomes were highly diverse and the highly virulent strains were phylogenetically closely related.

The genome of an intermediately virulent strain from a diseased pig, 89-1591 (serotype 2, ST25, ST27 Complex) is significantly different from the genome of the highly virulent GZ1, containing deletions of 169 genes and insertions of 387 genes [10]. The 89-1591 genome is 1,978,219 bp and contains 1,893 CDSs (the genome draft has 177 contigs). In this study we performed CGH on 39 isolates of *S. suis* from different clinical sources using the genome of *S. suis* 89-1591 as a reference. We also tested the capacity of 29 isolates to induce cytokine production, to resist phagocytosis *in vivo*, and to activate TLR2/6 signaling *in vitro* to assess correlation of genomic content variation with virulence variation.

## Materials and Methods

### Ethics statement

This study was reviewed and approved by the ethics committee of the National Institute for Communicable Disease Control and Prevention, Chinese Center for Disease Control and Prevention. The rights and the welfare of the mice used in the study were adequately protected. All necessary steps were taken to minimize suffering and distress to the mice in these studies.

### Strain selection and growth conditions

**Figure 1:**
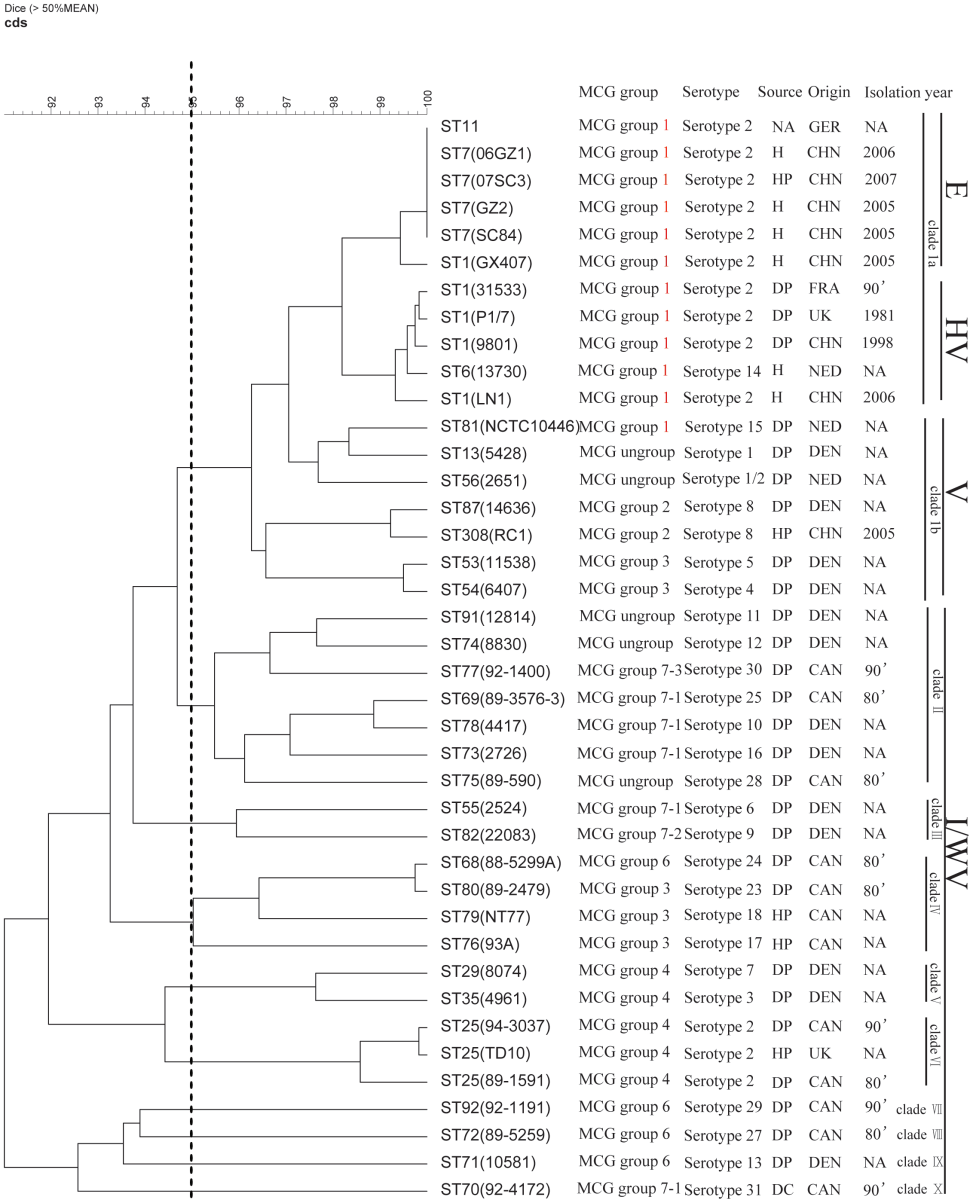
Phylogenetic relationship (UPGMA tree) of all 40 test strains and reference strain 89-1591 following DNA-DNA microarray hybridization. Absence and presence for each gene of each strain were translated to 0 and 1, respectively. The UPGMA tree shown was calculated from difference matrices with BioNumerics. E/HV: epidemic/highly virulent group, V: virulent group, I/WV: intermediately or weakly virulent group. CHN, China; NED, Netherlands; GER, Germany; CAN, Canada; FRA, France; DEN, Denmark, UK: united kingdom. DP, diseased pig; DC, diseased calf; HP, healthy pig; H, patient. NA, not applicable.

Thirty nine isolates of different serotypes, sources, geographic locations, isolation years, and STs were selected for this study to represent *S. suis* diversity, including six isolates from patients, 31 from pigs (four healthy pigs, 27 disease pigs), one from a calf, and one from an unknown host (Fig. 1). Of these, 26 were used in a previous NimbleGen microarray study [9]. To better evaluate the variation within *S. suis*, we added seven new isolates representing seven STs (ST13, ST29, ST68, ST70, ST72, ST92, and ST308), two ST25 strains (TD10 and 94-3037), two ST1 strains (9801 and GX407), and two ST7 strains (06GZ1 and 07SC3). These isolates were obtained in different years and from geographic locations. We included 11 isolates belonging to the ST1 complex with five from ST1, one from ST6, four from ST7, and one from ST11. These STs were considered as highly virulent strains in many studies[10], [11], [12], [13], [14]. ST7 possessed higher virulence levels than the ST1 strains[5], [10], [11]. ST7 and ST1 strains were defined as epidemic strains and highly virulent strains, respectively[10]. Four isolates belong to the ST27 complex with two ST25, one ST29, and one ST35 where the complex was considered less virulent [3], [6], [14]. To define the virulence levels accurately, we used epidemic (E), highly virulent (HV), virulent (V), intermediate and weakly virulent (I/WV) to represent the different levels. The virulence levels decreased incrementally from the E to I/WV

The *S. suis* strains were grown overnight on Columbia blood base agar (Guangzhou Detgerm Microbiological Science, P. R. China) at 37°C under CO_2_ conditions. For broth culture, isolated colonies were inoculated into 5 ml of Todd-Hewitt broth (THB, Oxoid Ltd., London, UK) and incubated for 8 h at 37°C with agitation (100 rpm) under CO_2_ conditions. Genomic DNA was extracted using a TIANamp Bacteria DNA Kit (Tiagen Biotech Co., Ltd, Beijing, P. R. China) according to the manufacturer's protocol. DNA samples were analyzed using agarose gel electrophoresis and quantified with a GeneQuant spectrophotometer *ND-1000* (NanoDrop, Wilmington, DE). All DNA samples were stored at −20°C in distilled H_2_O.

### DNA labeling

The genomic DNA samples (2 µg) of the test and reference strains were labeled Cy3- and Cy5- (TriLink Biotechologies) respectively using a randomly primed reaction according to the protocol of NimbleGen. Cy3 CPK 48 mer and Cy5 CPK 48 mer were purchased from IDT (5′ TTC CTC TCG CTG TAA TGA CCT CTA TGA ATA ATC CTA TCA AAC AAC TCA 3′; 250 nmole, HPLC purified).

### Microarrays and hybridization

The oligonucleotide probes of the microarray were synthesized *in situ* on a glass surface using the Maskless Array Synthesis (MAS) technology by NimbleGen (Madison, WI).

The entire genome of 89-1591 (GenBank accession number AAFA00000000, in draft) was represented on the glass slide by 17 unique “probe pairs” of 24-mer in situ-synthesized oligonucleotides. Each sample required a set of two microarrays, each consisting of about 380,000 probes. Five μg of labeled test and reference genomic DNA were hybridized to the arrays in 1× NimbleGen hybridization buffer (NimbleGen) for 16–20 h at 42°C. The microarrays were scanned at 5-nm resolution using a GenePix 4000b scanner (Axon Instruments, Union City, CA).

### Microarray data and statistical analysis

Hybridization signals represented as pixel intensities were extracted using NimbleScan v2.4 software (NimbleGen). Data were normalized and converted to estimates of abundance using the total signal intensity of the entire microarray to allow comparison of individual microarrays.

Determination of the presence or absence of genes was determined by comparing the hybridization signal strength of the test isolates to the reference strain 89-1591 using the method of Zheng *et al* [9]. Values derived from the 89-1591 genomic DNA hybridization were used as the baseline. We first computed the percentage of positive calls (PPC) in a gene which was equal to the percentage of positive call probes in the total number of tilling probes for a gene and a PPC of 50% or greater for ST25 strains and 92% or greater for other ST strains were used as the threshold to distinguish the presence of a gene.

### Comparative phylogenomic analysis of microarray data

All mobile genes were excluded from the analysis. The absence or presence of a gene was converted into a binary code (gene present in a given strain = 1, absent or highly divergent = 0) and analyzed using Cluster/TreeView. Trees based on the un-weighted-pair group method using average linkages (UPGMA) were constructed with BioNumerics (version 3.0; Applied Maths BVBA, Belgium). The correlations between these trees were calculated with BioNumerics.

### Measurement of blood bacterial loads and evaluation of cytokine production *in vivo*

C57BL mice (6 week, female) were injected intraperitoneally with 1×10^7^ cfu of live bacterial cells in 1 ml THB. Each isolate was injected into five mice. Mice injected with 1 ml THB only was used as the control group. At 8 h post-infection, the mice were sacrificed and peripheral blood was collected. For every100 µl blood, 10 µl of EDTA (125 mg/ml) was added to prevent clotting. Viable intracellular streptococci were determined using quantitative plating of serial dilutions of lysates on THB agar plates. Colonies were counted and expressed as cfu/ml. Serum was extracted from the blood. All serum samples were diluted at least four times before testing using the Bio-Plex Cytokine Reagent Kit (Bio-Rad, Hercules, CA). Cytokine levels were analyzed using the Bio-Plex Manager ™ 6.0 software. The experiments were repeated twice on different days.

### Activation of TLR2/6 signal *in vitro*

293-hTLR2/6 cells (InvivoGen, Toulouse, France) were used to determine the activation of TLR2/6. 293-hTLR2/6 cells were cultured according to the manufacturer's instructions.

Before the infection assays, 1.0 ml aliquots of cells (2×10^5^)/well were plated into 24-well flat-bottom plates (Becton Dickinson) and incubated in 5% CO_2_ at 37°C for 48 h to attain confluent growth (approximately 5.0×10^5^ cells/ml). *S. suis* (1×10^5^ cfu/well) was added to the cells and incubated for 24 h. Synthetic diacylated lipoprotein FSL-1 (0.1 µg/well, InvivoGen) was used as the positive control. Medium alone was used as the negative control. The supernatant was collected to measure IL-8 according to manufacturer's instructions (R&D Systems, Inc, Minneapolis, MN).

### Array data deposition

MIAME compliant array data from this study were deposited in the Gene Expression Omnibus database (http://www.ncbi.nlm.nih.gov/geo/) under GEO series accession number GSE40035. DNA sequence for gene CDS2157 obtained in this study was deposited in the Genebank (accession number JX978834).

### Statistics

The *in vitro* cytokine data were expressed as mean ± standard deviation for each strain. The *in vivo* cytokine values and bacterial counts were expressed as the median (interquartile range) for each strain. All data for each clade or group were expressed as the median (interquartile range). All statistical analyses of differences among clades or groups were performed using ANOVA on ranks. Differences were considered significant at a *p* value of <0.05.

## Results

### CGH and Phylogenomic analyses

Phylogenetic relationships inferred from the presence/absence of genes showed that 39 strains were divided into 10 clades at 95% similarity as the cut-off value (Fig. 1). Eighteen (46.1%) isolates were clustered into clade I containing 8 serotypes (serotype 1, 1/2, 2, 4, 5, 8, 14, and 15) and 11 STs (ST1, ST6, ST7, ST11, ST13, ST53, ST54, ST56, ST81, ST87, and ST308). The clade can be divided into two sub-clades: clade Ia (containing ST1, ST7, ST6, and ST11), and clade Ib (containing ST81, ST13, ST56, ST87, ST308, ST54, and ST53). Interestingly, ST81, ST87, ST56, ST53, and ST54 were closely related genetically to highly pathogenic strains in our previous study [9]. ST13 and ST308 were not in our previous study. Clades II to X contained one to seven STs which were all isolated from non-human sources.

### Differences in bacteremia levels of isolates from different clades

We selected ST7 (SC84) and ST1 (31533) to represent clade I a, 89-1591 to represent clade VI, and included all of the isolates from other clades to assay the differences in bacteremia levels. At 8 h post-infection, all mice were bacteremic and the test strains differed considerably in their ability to avoid bacterial killing *in vivo* (from 10^8^ to 10^2^).

**Figure 2:**
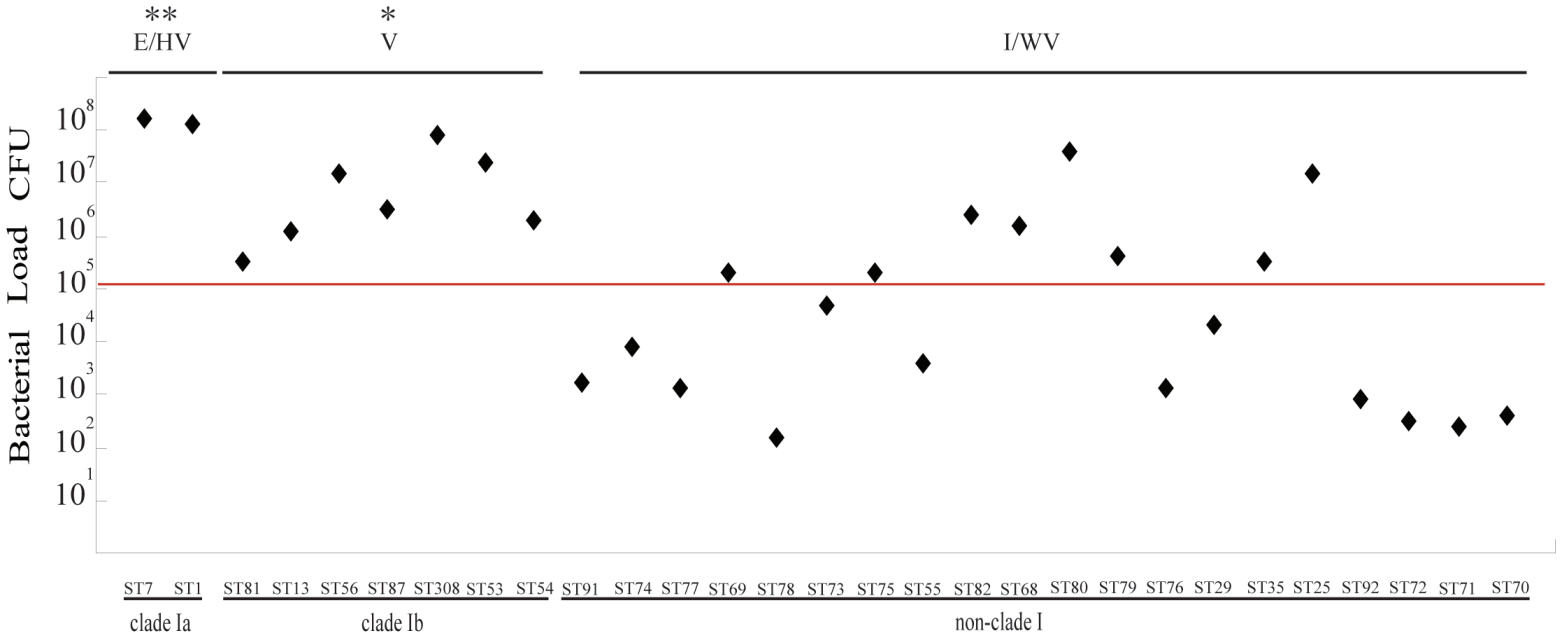
Blood bacterial load of the test strains in the infected mice. All data are from two independent experiments. E/HV: epidemic or highly virulent group, V: virulent group, I/WV: intermediately or weakly virulent group. *Significant, P<0.05, two asterisks (**): the value of group E/HV compared to group V and I/WV. One asterisk (*): the value of group V compared to group I/WV.

For 17 of 29 tested strains, the blood bacterial load in the mice infected was >10^5^, and for 12 of these 17 strains, blood bacterial load in mice infected was >10^6^ (Fig. 2). Interestingly of these 12 strains, the majority (eight) were from clade I. We observed significant differences in bacterial load between cladeI{1.6×10^7^ (2.3×10^6^–6.4×10^7^)} and non-cladeI{3.1×10^5^ (2.4×10^3^–1.1×10^7^)}. Within clade I, significant differences were observed between clade Ia strains {9×10^7^ (6.9×10^7^–1.2×10^8^)}and clade Ib strains {6.8×10^6^ (1.5×10^6^–3.8×10^7^)}.

### Difference of TNF-α and MCP-1 production *in vivo* induced by isolates from different clades

**Figure 3:**
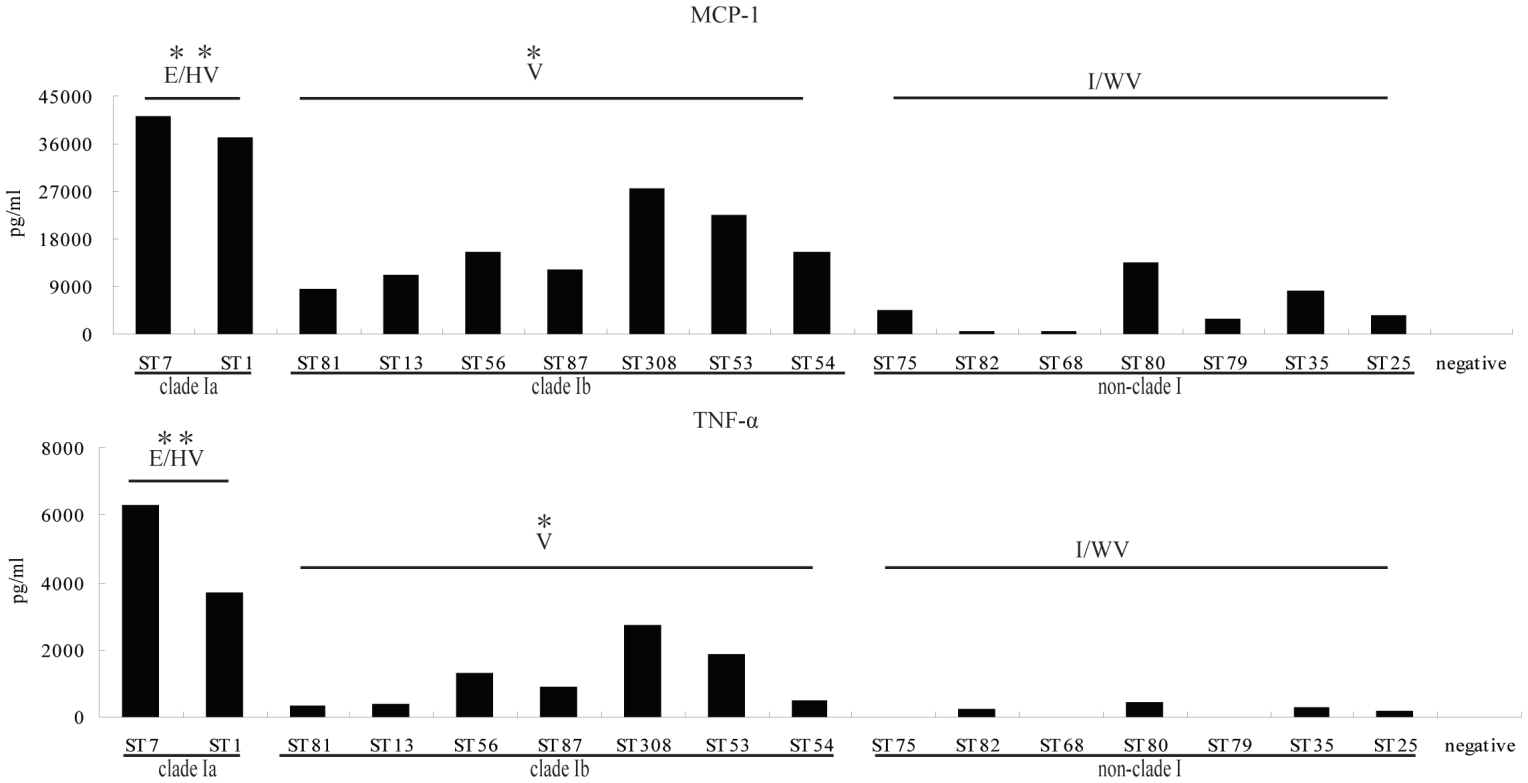
Production of MCP-1 and TNF-α by *S. suis in vivo*. All data are from two independent experiments. E/HV: epidemic or highly virulent group, V: virulent group, I/WV: intermediately or weakly virulent group. *Significant, P<0.05, two asterisks (**): the value of group E/HV compared to group V and I/WV. One asterisk (*): the value of group V compared to group I/WV.

Induction of TNF-α and MCP-1 by *S. suis* play an important role in the pathogenesis of *S. suis* [10], [11], [15]. Strains with blood bacterial loads <10^5^ did not induce obvious cytokine production (data not shown). Seventeen strains with blood bacterial loads >10^5^ were used to compare the capacity to induce the production of cytokine *in vivo* (Fig. 3). The concentrations of TNF-α in clade Ia, Ib and non-clade I were 4,119 pg/ml (3,268 pg/ml–6,862 pg/ml), 559 pg/ml (211 pg/ml–1,620 pg/ml) and 172 pg/ml (77 pg/ml–418 pg/ml), respectively. The concentration of MCP-1 in clade Ia, Ib and non-clade I was 35,684 pg/ml (30,019 pg/ml–40,383 pg/ml), 14,471 pg/ml (7,040 pg/ml–25,219 pg/ml) and 3,476 pg/ml (435 pg/ml–6,586 pg/ml), respectively. Production of TNF-α and MCP-1 in the mice infected with strains from clade I was statistically higher than observed in mice infected with strains from non-cladeI (*p*<0.05). Within clade I, strains from clade Ia possessed a higher capacity to induce cytokine production than strains from clade I b (*p*<0.05).

### Difference in activation of the TLR2/6 pathway by isolates from different clades

**Figure 4:**
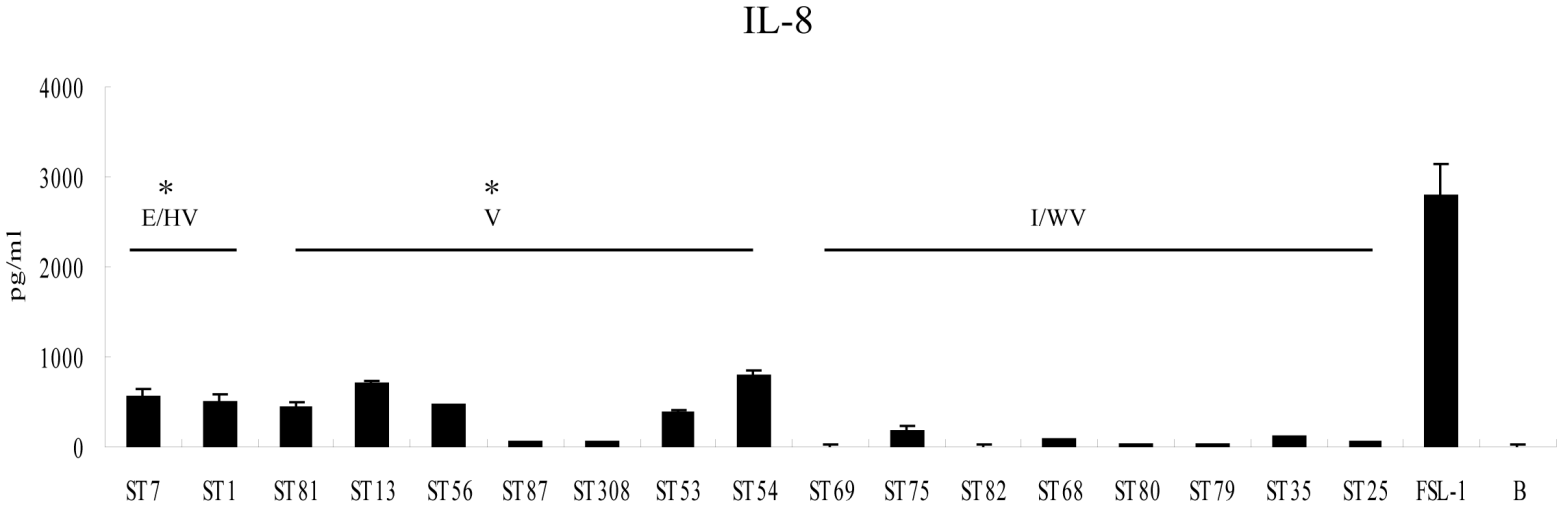
*S. suis* induced the production of IL-8 by 293-hTLR2/6 cells. All data are from three independent experiments. E/HV: epidemic or highly virulent group, V: virulent group, I/WV: intermediately or weakly virulent group, B: cell medium only. *Significant, P<0.05, asterisks: the value of group E/HV and V compared to group I/WV.

TLR2/6 plays an important role in inducing cytokine production by *S. suis* [15], [16], [17], [18]. We used 293-hTLR2/6 cells to investigate whether the 17 strains with >10^5^ blood bacterial load in mouse infections possessed different capacities to activate TLR2/6 signaling by determining the amount of IL-8 induced. The concentrations of IL-8 induced by clade I and non-clade I strains were 370 pg/ml (245 pg/ml–596 pg/ml) and 23 pg/ml (13 pg/ml–95 pg/ml), respectively (Fig. 4). The difference is statistically significant. The strains in clade Ia also induced higher levels of IL-8 at at 522 pg/ml (448 pg/ml–592 pg/ml) than clade Ib strains at 277 pg/ml (98 pg/ml–601 pg/ml), but the difference was not statistically significant.

### Core and Group specific genes

Of the 1,778 non-mobile genes surveyed, 1,087 genes (61.1% of the 1,591 genes on the microarray) were present in all of the test isolates, which is consistent with our previous report that used a different reference strain [9]. At the group level, 1,435 (80.7%), 1,333 (75.0%) and 1,100 (61.8%) of the genes were present in all isolates of the E/HV group, the V group and the I/WV group, respectively. Only one gene annotated as RNA binding S1 was specific to the I/WV group. Two genes were specific to the V group, one of which was annotated as a hypothetical protein DRAFT_0690 while the other was annotated as competence specific sigma factor, ComR2 [19]. Ninety-two genes were specific to the E/HV group (Table S1 and Table S2).

## Discussion

Different typing studies indicate that low virulent strains are more diverse than virulent strains where virulent strains possess closer genetic relationships and formed similar clusters, including AFLP, MLST and microarray[9], [20], [21], [22]. Wu, Z et al showed the classification of tested strains based on CGH was in good agreement with the differential virulence of these strains. CGH was shown to be valuable in evaluating the virulence of *S. suis* [22].

Combining experimental data with phylogenetic relationships, we can divide the isolates into three groups: epidemic or highly virulent group (E/HV, clade I a), virulent group (V, clade I b), and intermediatly or weakly virulent group (I/WV, non-clade I). We showed the virulence levels of the three groups decreased incrementally from the E/HV group to the I/WV group. This hypothesis is partially supported by a previous report that the virulence of an ST13 strain was higher than an ST82 strain and lower than an ST1 strain using experimental infections in pigs [21].

In our previous study, we developed a method to define the minimum core genome of *S. suis* strains. Population genetic analysis of the MCG SNPs divided the *S. suis* population into seven core genome groups (MCG group 1 to 7). MCG group 1 included all of the highly virulent isolates of ST1 and the epidemic isolates of ST7, which contained the greatest number of virulence genes. MCG group 2, 3, and 4 carried fewer numbers of virulence genes than MCG group 1. MCG group 6 carries the lowest number of virulence genes. MCG group 7 carries slightly more virulence genes than MCG group 6 does and obviously less than MCG group 2, 3 and 4[23].

Here the E/HV group contained the MCG group 1 strains. The V group contained one MCG group 1(ST81)strain, two MCG group 2 strains (ST308 and ST87), two MCG group 3 strains (ST54 and ST53) and two MCG un-grouped strains (ST13 and ST56). The I/WV group was from MCG groups 3, 4, 6, 7 and un-grouped. Note ST76, ST79, and ST80 (MCG group 3) were also not clustered in the same clade with ST53 and ST54 and were more related to MCG group 4 strains based on population structure and distribution of regions of difference (RDs) in our previous study [9].

Comparing to the phylogenetic tree obtained in our previous[9] and present study, there was consistency in major branching orders of strains from the E/HV and V groups in both studies. The differences between them were primarily observed in strains from the I/WV group known to be diverse. Clade II and clade III were genetically close to clade Iin the present study, but were opposite in the previous study.

In addition to classical evolutionary change via single site substitutions, bacterial genomes evolve rapidly by gaining new sets of genes or by losing an existing set of genes. *S. suis* ST25 was located at an early stage in the evolution of *S. suis*, compared to ST1 and ST7[10], [23]. When ST1/ST7 strain was used as a reference strain, the genes gained during evolution contributed greatly to the phylogenetic tree. When ST25 strain was used as the reference strain, the lost genes contributed greatly to the phylogenetic tree. The difference in weighing of two kinds of genes in the formation of the tree may contribute to the inconsistency. Not having a complete reference genome used in the present study may also partially contribute to the differences.

Resistance to phagocytosis and persistence *in vivo* at high concentrations is necessary to induce the inflammatory consequences of *S. suis* [24]. In the 29 tested strains, the blood bacterial load of the mice infected with 12 strains was >10^6^. Eight came from the E/HV and V group. The number of bacteria being internalized in the E/HV and V group was less than the I/WV group. The number of bacteria being internalized in the E/HV group was less than the V group. The data suggest a delayed capacity to establish infection or higher susceptibility to be cleared by the host contributed partially to the decrease in cytokine production *in vivo*.

Some strains within the I/WV group persist *in vivo* at high concentrations, such as ST80 and ST25. This may be due to several reasons.

CPS, especially for sialic-acid containing capsules, played a critical role in *S. suis* to avoid phagocytic killing. The CPS of ST25 strain (89-1591 serotype 2) contains sialic-acid. The structure or component of serotype 2 CPS may contribute to the high bacterial load of ST25 strain *in vivo* [25], [26].ST80 strain possessed the *sly* gene. Suilysin may help *S. suis* evade innate immunity by decreasing the phagocytosis by neutrophils[27]. Phagocytosis related receptors on the host cell also should be mentioned. In our un-submitted data, ST25 strain possessed higher capacity to resist the phagocytosis by microphage cell line (RAW264.7) than microglia cell line (BV2). ST56, ST53, and ST308 possessed higher capacity to resist the phagocytosis by BV2 cells than ST25. In future studies, the bacterial load in different organs should be investigated.

TLR2/6 is involved in the recognition of *S. suis* by the immune system[15], [16], [17], [18]. Here we found there was a correlation between the capacity to induce TLR2/6 signaling and the virulence level. Although the capacity to resist phagocytosis by ST82, ST68, ST80, and ST25 was similar to strains of the V group, the low level of TLR2/6 activation induced by them may partially explain why less cytokines were produced *in vivo*. We did not observe a statistical difference in TLR2/6 activation between the E/HV and V groups that may differ in inducing other inflammation pathways.

Use of the genome from a less virulent strain as reference allow identifications that may be negatively associated with virulence. In this study, we found that a CDS2157 annotated as RNA binding S1 (2,157 bp) within cont213 was a gene specific to the I/WV group. This gene was present in all of V and I/WV strains except in ST13 strain but not in the E/HV group. CDS2157 belongs to the Tex family proteins that contain an S1 RNA-binding domain at the C-terminus. Members of this family are putative transcriptional accessory factors. CDS2157 has the closest similarity to the *tex* gene in *Streptococcus salivarius* with an overall nucleotide sequence homology of 60.5% (Sequence ID: gb|CP002888.1|). The *tex* gene plays a role in toxin expression in *Bordetella pertussis* and in *C. perfringens* [28]. However, the Tex of *Streptococcus pneumoniae* has no regulatory function in the expression of the pneumococcal major toxin pneumolysin [29]. The role of this gene in the pathogenesis of *S. suis* requires further study.

The majority (70) of the E/HV specific genes consist of two or more contiguous genes in blocks ranging from 0.7 to 14.6 kbp in size. We defined them as genomic islands (GIs). We proposed that the specific genes grouped together as chromosomal blocks which have been acquired in the reference genome by horizontal transfer.

In our previous study, we found eight regions of differences (RDs) preferentially associated with highly pathogenic strains[9]. In the present study, only partial genes of RD15 and RD 17 were found in the E/HV group specific genes.

Two genes in RD15 were found in the E/HV specific genes and were defined as GI 10. RD15, encoding a *srtF* pilus cluster, has recently been reported to be preferentially distributed in ST1 clonal complex strains[30]. The annotations of two genes were signal peptidase IB and Ribonucleases G and E, respectively.

Eleven genes in RD17 were found in E/HV specific genes. These genes consisted GI 11 and GI 14. RD17, encoding serotype 2 capsular polysaccharide, are known virulence factors of *S. suis*. All strains but ST6 (serotype 14) in E/HV group were serotype 2.

Two E/HV specific genes consisted GI 7. One of them encoded *mrp* variation, which some frameshift mutations were found comparing to *mrp* harbored by strains from E/HV group. The presence of the MRP gene did not always correlate with actual expression of the respective protein. Nonsense mutations at 2.1 kb from the ATG initiator codon and frameshift mutations at different short distances from the reported initiator ATG resulted in negative for MRP by Western-blot[31]. The resolution ratio of microarray was not high enough to discriminate *mrp* gene variant from *mrp*. The conclusion that mrp was not significantly associated with strains from E/HV group should be re-checked in our previous study. Further research is needed to investigate whether the isolates express MRP protein.

Carbohydrate transport genes and cell wall/membrane biogenesis genes were the category significantly represented by 92 specific genes. Carbohydrate transport genes may have no role in pathogenesis but are likely to be a reflection of adaptation to the oral and respiratory tract environments which may contribute to the pathogenic potential. Cell wall/membrane biogenesis genes may contribute to evade innate immunity by decreasing the phagocytosis *in vivo*.

In summary, we show that *S. suis* strains were classified into three groups using genomic differences with different levels of pathogenicity. Our study provided further insight into the genomic and virulence variation of *S. suis*.

## Supporting Information

Table S1 Core genes and specific core genes for each group.
(XLS)

Table S2 Summary of the 17 GI features.
(DOC)
