# Supplementary material for: Comparative Genomic Hybridization Identifies Virulence Differences in Streptococcus suis
Source: PLoS One. 2014 Feb 4;9(2):e87866. doi: 10.1371/journal.pone.0087866 (PMC3913679; doi:10.1371/journal.pone.0087866)
Supplement: Table S2 — Summary of the 17 GI features. (DOC) [file pone.0087866.s002.doc]

**Table S2 Summary of the 17 GI features.**

| **RD** | **Position in 89-1591 genome** | | **Length (bp)** | **GC content (%)** | **Virulence-related and other notable factors encoded** |
| --- | --- | --- | --- | --- | --- |
| **GI1** | 2662201_Cont265 | 33689..48265 | 1477 | 33.74 |  |
| **GI2** | 2662201_Cont268 | 11049..13792 | 2744 | 36.84 |  |
| **GI3** | 2662201_Cont262 | 26603..35672 | 9070 | 39.76 |  |
| **GI4** | 2662201_Cont259 | 26692..27713 | 1022 | 35.91 |  |
| **GI5** | 2662201_Cont259 | 28584..31069 | 2486 | 40.43 |  |
| **GI6** | 2662201_Cont258 | 4164..5293 | 1130 | 39.91 |  |
| **GI7** | 2662201_Cont258 | 15273..20020 | 4748 | 39.66 | *mrp* variation |
| **GI8** | 2662201_Cont252 | 1878..3134 | 1257 | 40.25 |  |
| **GI9** | 2662201_Cont243 | 3206..16348 | 13143 | 42.65 |  |
| **GI10** | 2662201_Cont245 | 3104..7871 | 4768 | 39.22 | SrtF pilus |
| **GI11** | 2662201_Cont233 | 194..4007 | 3814 | 37.47 | CPS2 synthesis |
| **GI12** | 2662201_Cont241 | 1992..6308 | 4317 | 41.58 |  |
| **GI13** | 2662201_Cont225 | 5360..6650 | 1291 | 36.48 |  |
| **GI14** | 2662201_Cont151 | 688..7147 | 6460 | 39.40 | CPS2 synthesis |
| **GI15** | 2662201_Cont140 | 1664..2376 | 713 | 36.89 |  |
| **GI16** | 2662201_Cont191 | 72..5028 | 4957 | 38.01 |  |
| **GI17** | 2662201_Cont99 | 638..1555 | 918 | 34.1 |  |
